# Comparison of three risk stratification scores in gastroschisis neonates: gastroschisis prognostic score, gastroschisis risk stratification index and complex gastroschisis

**DOI:** 10.1007/s00383-022-05180-5

**Published:** 2022-07-26

**Authors:** Asta Tauriainen, Arimatias Raitio, Tuomas Tauriainen, Kari Vanamo, Ulla Sankilampi, Ilkka Helenius, Anna Hyvärinen

**Affiliations:** 1 Department of Pediatric Surgery, University of Eastern Finland and Kuopio University Hospital, Puijonlaaksontie 2, 70210 Kuopio, Finland; 2 Department of Pediatric Surgery, University of Turku and Turku University Hospital, Turku, Finland; 3 Department of Surgery, Oulu University Hospital, Oulu, Finland; 4 Department of Pediatrics, Kuopio University Hospital, Kuopio, Finland; 5 Department of Pediatrics, School of Medicine, University of Eastern Finland, Kuopio, Finland; 6 Department of Orthopedics and Traumatology, University of Helsinki and Helsinki University Hospital, Helsinki, Finland; 7 Department of Pediatric Surgery, University of Tampere and Tampere University Hospital, Tampere, Finland

**Keywords:** Gastroschisis, Risk stratification, Complex gastroschisis, Score, Level of evidence: Level III

## Abstract

**Purpose:**

The aim of the study was to compare and evaluate the utility of three different risk stratification scores for gastroschisis neonates; simple/complex gastroschisis, gastroschisis prognostic score and risk stratification index.

**Methods:**

Data of neonates born with gastroschisis between the years 1993 and 2015 were collected. The national registers and patient records of four Finnish University Hospitals were retrospectively reviewed. Logistic and linear regression analysis were performed to identify independent predictors for adverse outcomes. The efficacy of these prognostic methods was further assessed using ROC-curves and DeLong (1988) test.

**Results:**

Gastroschisis risk stratification index was an acceptable predictor of in-hospital mortality, AUC 0.70, 95% CI 0.48–0.91, *p* = 0.049. Complex gastroschisis and gastroschisis prognostic score were able to predict short bowel syndrome, AUC 0.80, 95% CI 0.58–1.00, *p* = 0.012 and AUC 0.80, 95% CI 0.59–1.00, *p* = 0.012, respectively.

**Conclusion:**

There are three easily obtainable risk stratification scores for outcome prediction in gastroschisis patients, however, their predictive ability did not have a statistical difference in the present study. The Gastroschisis risk stratification index seemed to perform moderately well in mortality prediction.

## Introduction

Gastroschisis is a congenital defect of the anterior abdominal wall, characterized by prolapse of intestine and other abdominal organs outside the abdominal cavity. Surgical correction is required soon after delivery. The prevalence of gastroschisis is increasing worldwide and has been reported to be 1 to 5 in 10000 live births. [1–3]. Survival rates have been published to be greater than 90% [2, 4, 5]. The negative impact of additional intestinal anomalies or bowel compromise have described in the literature and division to simple and complex gastroschisis (complicated by atresia, perforation, necrosis and/or volvulus) is the most common categorization method [6, 7]. Cowan and colleagues introduced a Gastroschisis Prognostic Score (GPS) in 2012, which is a risk stratification method based on visual assessment of bowel appearance after delivery. It includes the following variables: matting, necrosis, atresia and perforation. [8]. Elevated scores have been shown to associate with increased length of stay, increased total time of parenteral nutrition, increased time to enteral feeds and mortality [8] in addition to complications [9]. Another risk score for gastroschisis is the Gastroschisis Risk Stratification Index (GRSI), which includes intestinal atresia, necrotizing enterocolitis, rare cardiac anomalies and lung hypoplasia to identify patients at greatest risk for mortality. This method was shown to successfully identify infants at a high risk for death. [10]. To our knowledge, studies comparing multiple risk assessment modalities for gastroschisis are scarce in the current literature. These scoring systems could provide valuable information to both clinicians and researchers.

The aim of the study was to compare and evaluate the utility of three different risk stratification scores for gastroschisis neonates; simple/complex gastroschisis, GPS and GRSI.

## Materials and methods

### Patients

We conducted a retrospective study of neonates born with gastroschisis and treated at the Pediatric Surgery Departments of four University hospitals, Tampere, Turku, Kuopio and Oulu, in Finland from 1993 to 2015. Unfortunately, we do not have data from the fifth university hospital Helsinki, which would have made the database nationwide. Gastroschisis patients were identified from the hospital registers based on the diagnoses according to International Classification of Diseases (ICD-9 code 756.73, after the year 1994 Q79.3 ICD-10 code). Data regarding maternal factors, prenatal observations, initial presentation at birth, surgical treatment, post-operative treatment, complications and short-term outcomes were collected from the patient files at each hospital, the Finnish Medical Birth Register and the Finnish Register of Congenital Malformations maintained by the Finnish Institution for Health and Welfare. The mean follow-up time of this database was 11 years. The institutional review boards of the participating hospitals and the Finnish Institution for Health and Welfare accepted the study. The need for patients’ written consent was waived.

### Definitions

The birth weight z score was obtained using the contemporary Finnish Birth size reference [11]. Small for Gestational Age (SGA) was defined as birth weight below – 2 Standard deviation. Organ prolapse was defined as any intra-abdominal organ fully or partially protruding trough the fascial defect at birth, excluding small and large intestine. Patients with associated bowel atresia, bowel perforation, bowel necrosis or volvulus were defined to have a complex gastroschisis, as suggested by Molik and Abdullah [6, 12]. GRSI includes a scoring system for intestinal atresia, necrotizing enterocolitis, rare cardiac anomalies and pulmonary hypoplasia with range of 0–10 points. Atresia gives 1, NEC 2, rare cardiac anomalies 3 and pulmonary hypoplasia 4 points [10]. Rare cardiac anomalies were selected according to Arnold et al. Points of GPS range from 0 to 12 and variables are matting, atresia, perforation and necrosis [8, 9]. Descriptions of bowel injury were based on the study and instructions for use by Cowan et al. [13]. Points of matting are 0 for none, 1 for mild and 4 for severe. Absent atresia, perforation or necrosis values 0, suspected atresia 1, present atresia or perforation 2 and present necrosis 4.

### Outcome endpoints

The primary endpoints of the present study were to compare and evaluate the function of three different risk stratification scores for gastroschisis neonates in Finland; simple/complex gastroschisis, GPS and GRSI. Our outcomes of interest were in-hospital mortality, short bowel syndrome, positive blood culture, intravenous nutrition time, in-hospital stay in days and a re-laparotomy for any of the following—perforation, ischemia or necrosis of the bowel.

### Statistical analysis

Statistical analysis was performed using SPSS software (version 25.0, IBM Corporation, New York, USA). No attempt to replace missing values was made. Categorical variables are reported as counts and percentages. Continuous variables are reported as means and standard deviations. Logistic and linear regression analysis were performed to identify independent predictors for adverse outcomes. The following variables were included in the multivariate models: gestational age, birth weight, Apgar score at 5 min of age, gender, hospital transfer after delivery, prenatal diagnose known, any organ prolapse other than bowel and twin status in addition to each risk stratification method individually. Receiver operating characteristic curves (ROC) were used to assess the discriminatory power of GPS, GRSI and simple/complex gastroschisis. ROC is a quantitative measure of the ability of a diagnostic tool to differentiate between dichotomous events. Area under the curve (AUC) values were calculated to compare these models in a free web-based application [14] with DeLong (1988) test [15]. All statistical tests were performed as two-tailed and a *p* value ≤ 0.05 represented statistical significance. AUC values depicting the accuracy of risk stratification were classified as fail (0.50–0.59), poor (0.60–0.69), moderate (0.70–0.79), good (0.80–0.89) and excellent (0.90–1.00).

## Results

A total of 156 newborns with gastroschisis were born between January 1993 and December 2015. Thirteen neonates were excluded, one who received most of the treatment in a non-participating center, one with missing patient records and 11 not having all variables needed to measure the scores. Altogether 143 neonates were included in the study. Perinatal characteristics are summarized in Table 1. There were 79 male (55.2%) and 64 female infants. Gastroschisis was prenatally diagnosed in 123 cases (86.0%). Ten patients (7.0%) were born in a central hospital and required transfer to a University Hospital postnatally. The average birth weight and gestational age were 2530 ± 564 g and 36.7 ± 1.8 weeks, respectively. Vaginal delivery rate was 31.5 percent. Nine infants died before discharge from hospital (6.3%). Re-laparotomy for bowel perforation, ischemia or necrosis was performed for seven infants (4.9%). Short bowel syndrome was diagnosed in 6 cases (4.2%) and 21 infants had a positive blood culture (14.7%). The mean duration of parenteral nutrition and hospital stay were 55.3 ± 198.4 and 39.2 ± 50.0 days, respectively.

**Table 1:** Overall demographic data

Variable	Overall series *n* = 143
Gestational age in weeks	36.7 ± 1.8
Weight in grams	2530.0 ± 563.6
Weight < 2500 g	77 (53.8%)
SGA	53 (37.1%)
Prematurity < 37 weeks	77 (53.8%)
Male	79 (55.2%)
Twin	6 (4.2%)
Vaginal birth	45 (31.5%)
Hospital transfer at birth	10 (7.0%)
Organ prolapse other than bowel	71 (49.7%)
Apgar score at 5 min age	7.9 ± 1.8

Nominal variables are presented as counts and percentages, continuous variables as a mean and standard deviation

*SGA* small for gestational age

Numbers of infants classified into each risk prediction model are presented in Table 2. Comparison of risk prediction models in patients with gastroschisis are presented in Tables 3 and 4. ROC-curves were drawn for dichotomized variables. GPS was found to reliably predict short bowel syndrome, AUC 0.80, 95% CI 0.59–1.00, *p* = 0.012, as was also classification to complex gastroschisis, AUC 0.80, 95% CI 0.58–1.00, *p* = 0.012. GRSI was a moderate predictor of in-hospital mortality, AUC 0.70, 95% CI 0.48–0.91, *p* = 0.049. Furthermore, we conducted multivariate models adjusted for relevant perinatal variables and beforementioned risk scores. Gastroschisis prognostic score was found to be an independent predictor for short bowel syndrome, OR 1.63, 95% CI 1.07–2.50, *p* = 0.023. Gastroschisis risk stratification index was an independent predictor for re-laparotomy for perforation, ischemia, necrosis and also for in-hospital death, OR 4.27, 95% CI 1.40–13.07, *p* = 0.011 and OR 6.38 95% CI 1.66–24.51, *p* = 0.007, respectively. Complex gastroschisis was associated with increased length of stay, *p* = 0.000.

**Table 2:** Numbers of infants classified into each risk prediction models

Score points	GPS	GRSI	Complex gastroschisis
0	67 (46.9%)	129 (90.2%)	123 (86.0%)
1	37 (25.9%)	10 (7.0%)	20 (14.0%)
2	3 (2.1%)	2 (1.4%)	
3	3 (2.1%)	1 (0.7%)	
4	20 (14.0%)	1 (0.7%)	
5	3 (2.1%)	0 (0%)	
6	5 (3.5%)	0 (0%)	
7	0 (0%)	0 (0%)	
8	2 (1.4%)	0 (0%)	
9	1 (0.7%)	0 (0%)	
10	2 (1.4%)	0 (0%)	
11	0 (0%)		
12	0 (0%)		

Gastroschisis prognostic score (GPS) ranges between 0 and 12. The degree of intestinal matting, atresia, perforation and necrosis are assessed in GPS. Points for matting are 0 for none, 1 for mild and 4 for severe. Absent atresia, perforation or necrosis gives a value of 0, suspected atresia 1, present atresia or perforation 2 and present necrosis 4 points. Gastroschisis risk stratification index (GRSI) includes a scoring system assessing intestinal atresia, necrotizing enterocolitis (NEC), rare cardiac anomalies and pulmonary hypoplasia with a range of 0–10 points. Atresia gives 1, NEC 2, rare cardiac anomalies 3 and pulmonary hypoplasia 4 points. In complex (1) versus simple (0) gastroschisis, patients with associated bowel atresia, bowel perforation, bowel necrosis or volvulus were defined as having a complex gastroschisis. Nominal variables are presented as counts and percentages

*GPS* gastroschisis prognostic score, *GRSI* gastroschisis risk stratification index

**Table 3:** The numbers of outcomes between gastroschisis prognostic score, Gastroschisis risk stratification index and complex gastroschisis scoring systems

Score points	0	1	2	3	4	5	6	7	8	9	10	11	12
Gastroschisis prognostic score (0–12)													
In-hospital mortality	4 (6.0)	2 (5.4)	1 (33.3)	0	0	1 (33.3)	0	0	1 (50.0)	0	0	0	0
Re-laparotomy for perforation/necrosis	3 (4.5)	1 (2.7)	0	1 (33.3)	0	1 (33.3)	0	0	1 (50.0)	0	0	0	0
Re-laparotomy for occlusion	1 (1.5)	5 (13.5)	0	1 (33.3)	0	0	2 (40.0)	0	2)100.0)	1 (100.0)	2 (100.0)		
Short bowel syndrome	1 (1.5)	0	0	1 (33.3)	1 (5.0)	0	1 (20.0)	0	1 (50.0)	0	1 (50.0)	0	0
Positive blood culture	11 (17.7)	6 (16.7)	0	2 (66.7)	1 (5.3)	0	0	0	1 (100.0)	0	0	0	0
Parenteral nutrition time in days	55.1 ± 249.4	24.6 ± 17.4	41.0 ± 9.5	193.0 ± 210.7	23.5 ± 12.4	14.3 ± 12.1	243.2 ± 417.5	N/A	322.5 ± 447.6	N/A	N/A	N/A	N/A
In-hospital stay in days	36.4 ± 51.9	30.0 ± 18.0	58.3 ± 18.5	148.7 ± 170.1	32.0 ± 17.0	18.0 ± 17.3	90.0 ± 78.3	N/A	101.5 ± 135.1	N/A	N/A	N/A	N/A
Gastroschisis risk stratification index (0–10)													
In-hospital mortality	5 (3.9)	1 (10.0)	1 (50.0)	1 (100.0)	1 (100.0)	0	0	0	0	0	0		
Re-laparotomy for perforation/necrosis	4 (3.1)	1 (10.0)	2 (100.0)	0	0	0	0	0	0	0	0		
Re-laparotomy for occlusion	6 (4.7)	4 (40.0)	1 (50.0)	1 (100.0)	1 (100.0)	0	0	0	0	0	0		
Short bowel syndrome	3 (2.3)	3 (30.0)	0	0	0	0	0	0	0	0	0		
Positive blood culture	20 (16.7)	1 (11.1)	0	0	0	0	0	0	0	0	0		
Parenteral nutrition time in days	42.7 ± 178.8	244.8 ± 363.9	14.5 ± 16.3	N/A	N/A	N/A	N/A	N/A	N/A	N/A	N/A		
In-hospital stay in days	36.3 ± 47.8	93.6 ± 61.3	17.5 ± 20.5	N/A	N/A	N/A	N/A	N/A	N/A	N/A	N/A		
Complex gastroschisis (simple vs. complex)													
In-hospital death	6 (4.9)	3 (15.0)											
Re-laparotomy for perforation/necrosis	4 (3.3)	3 (15.0)											
Re-laparotomy for occlusion	6 (4.9)	5 (25.0)											
Short bowel syndrome	1 (0.8)	4 (25.0)											
Positive blood culture	19 (16.2)	2 (12.5)											
Parenteral nutrition time in days	40.5 ± 181.5	146.2 ± 270.0											
In-hospital stay in days	33.2 ± 39.7	78.2 ± 84.2											

Gastroschisis prognostic score (GPS) ranges between 0 and 12. The degree of intestinal matting, atresia, perforation and necrosis are assessed in GPS. Points for matting are 0 for none, 1 for mild and 4 for severe. Absent atresia, perforation or necrosis gives a value of 0, suspected atresia 1, present atresia or perforation 2 and present necrosis 4 points. Gastroschisis risk stratification index (GRSI) includes a scoring system assessing intestinal atresia, necrotizing enterocolitis (NEC), rare cardiac anomalies and pulmonary hypoplasia with a range of 0–10 points. Atresia gives 1, NEC 2, rare cardiac anomalies 3 and pulmonary hypoplasia 4 points. In complex (1) versus simple (0) gastroschisis, patients with associated bowel atresia, bowel perforation, bowel necrosis or volvulus were defined as having a complex gastroschisis. Nominal variables are presented as counts and percentages, continuous variables as a mean and standard deviation

*N/A* not available

**Table 4:** The predictive abilities of the risk scores

Outcome	Multi-variate *p* value for the risk score^α^	AUC and 95% confidence interval	AUC *p*-value^β^
Gastroschisis prognostic score			
In-hospital mortality	0.582	0.531 95% CI 0.326–0.737	0.755
Re-laparotomy for perforation/necrosis	0.932	0.568 95% CI 0.325–0.811	0.546
Re-laparotomy for occlusion	0.053	0.731 95% CI 0.587–0.875	0.011*
Short bowel syndrome	0.023*	0.804 95% CI 0.585–1.000	0.012*
Positive blood culture	0.276	0.446 95% CI 0.318–0.574	0.431
Parenteral nutrition time in days	0.864	N/A	
In-hospital stay in days	0.081	N/A	
Gastroschisis risk stratification index			
In-hospital mortality	0.007*	0.697 95% CI 0.479–0.914	0.049*
Re-laparotomy for perforation/necrosis	0.011*	0.680 95% CI 0.437–0.923	0.109
Re-laparotomy for occlusion	0.074	0.690 95% CI 0.500–0.881	0.036*
Short bowel syndrome	0.315	0.703 95% CI 0.454–0.952	0.094
Positive blood culture	0.314	0.469 95% CI 0.340–0.599	0.657
Parenteral nutrition time in days	0.782	N/A	
In-hospital stay in days	0.690	N/A	
Complex gastroschisis			
In-hospital death	0.274	0.603 95% CI 0.393–0.813	0.301
Re-laparotomy for perforation/necrosis	0.189	0.652 95% CI 0.416–0.888	0.176
Re-laparotomy for occlusion	0.172	0.670 95% CI 0.481–0.860	0.061
Short bowel syndrome	0.036*	0.862 95% CI 0.686–1.000	0.003*
Positive blood culture	0.245	0.485 95% CI 0.352–0.618	0.829
Parenteral nutrition time in days	0.354	N/A	
In-hospital stay in days	0.000*	N/A	

*AUC* area under the curve, *CI* confidence interval

^α^Statistical significance of the risk score in logistic/linear regression model

^β^Statistical significance of the produced area under the curve when the null hypothesis is: AUC = 0.5

^*^Represents statistical significance

We compared the areas under the ROC-curves of GPS, GRSI and complex gastroschisis with DeLong (1988) test. There were no significant differences between these risk stratification scores in any of the outcome endpoints (in-hospital mortality, re-laparotomy for perforation, ischemia, necrosis, short bowel syndrome, positive blood culture, intravenous nutrition time and in-hospital stay in days).

## Discussion

The present study demonstrates the function of three different risk stratification scores. Our comparison analysis shows that their ability to predict specific outcome measures differed from each other. In our study cohort, only GRSI was an independent predictor for in-hospital mortality, but not the other studied outcome measures. Gastroschisis prognostic score and complex gastroschisis classifications were only able to predict reliably short bowel syndrome.

The literature has provided evidence that there are different types of gastroschisis with varying clinical outcomes of morbidity and mortality [6, 7, 16, 17]. Our results are in line with these studies.

Molik et al. and Arnold et al. showed in their studies with 103 and 4344 gastroschisis infants that cases with complex gastroschisis had decreased survival [6, 18]. Complex gastroschisis was significantly associated with death according to Arnold et al. (2007) [18]. Molik et al. reported 32 (31.0%) complex cases in their study. All deceased patients were classified as complex, which resulted in a mortality rate of 28.0% among this patient group [6]. The incidence of complex gastroschisis reported by Arnold et al. was 474 cases (10.9%) [18], which is similar to our finding of 20 complex cases out of 143 gastroschisis infants (14.0%). Their study reported that coexisting bronchopulmonary dysplasia had a higher prevalence in the complex group. Moreover, cardiac diseases were more common in this patient population. [18]. Furthermore, Laje et al. evaluated outcomes and complications during initial hospitalization of 125 simple and 58 complex gastroschisis cases. There was no neonatal mortality. [19]. Complex gastroschisis has been associated with longer mechanical ventilator time [6], parenteral nutrition time and in-hospital stay [6, 19].

GPS and complex gastroschisis classifications are based on bowel appearance after delivery [6–8, 18]. In the study by Cowan et al., the interobserver correlation with GPS risk score calculation was shown to be reliable between different surgeons [8]. On the contrary, utilization of the GRSI score requires additional examinations, such as ultrasound or additional postnatal imaging [10], which prevents the score from being used at bedside or immediately after birth.

Arnold et al. developed GRSI to predict death in gastroschisis using data consisting of 4310 patients with gastroschisis from 1988 to 2002. They divided the patients into two subgroups according to the risk of mortality. High-risk patients have scores greater or equal to 3 and low-risk patients fewer or equal to 2. Infants classified in the high-risk group had greater than a 10% risk of death compared to the low-risk group, with an overall mortality of 3.0% [18]. Interestingly, there was a statistically significant difference between mortality rates of high-risk group (GRSI) and complex gastroschisis group [7], 29.4% vs. 9.6%, respectively, *p* = 0.02.

Indeed, it could be speculated that the small number of patients and the relatively long follow-up period in this study could introduce a bias based on the improvement of care over time. However, the treating hospital or the time-frame of birth did not have an effect on mortality in a study by Tauriainen et al. (2021), which was based mostly on the same database [20]. On this basis, the results of the present study can be considered reliable.

The limitations of present study include the retrospectively collected data with some missing values and relatively small sample size. We speculate that the small number of patients in our high-risk groups might have prevented us from obtaining significant results. We do not have photographs of the appearance of protruding intestines or organs. The risk scoring was performed using only electronic and paper patient records. In addition to before mentioned scores, Score for Neonatal Acute Physiology, Version II (SNAP-II) and Score for Neonatal Acute Physiology, Perinatal Extension, Version II (SNAPPE-II), are also designed for measurement of illness severity for all neonates requiring intensive care. SNAP-II is based on six different vital signs and laboratory tests and SNAPPE-II adds to points for to birth weight, low Apgar score and small for gestational age (SGA) [21]. However, we were unable to utilize SNAP-II or SNAPPE-II scoring methods due to lack of data.

## Conclusions

In conclusion, there are three easily obtainable risk stratification scores for outcome prediction in gastroschisis patients with different predictive profiles. However, their predictive ability did not have a statistical difference. The Gastroschisis risk stratification index performed moderately well in mortality prediction, whereas gastroschisis prognostic score and complex gastroschisis were able to identify reliably patients suffering from short bowel syndrome. Further studies with larger databases are needed to compare these risk scores in more detail.
